# Overexpression of a Gene Involved in Phytic Acid Biosynthesis Substantially Increases Phytic Acid and Total Phosphorus in Rice Seeds

**DOI:** 10.3390/plants4020196

**Published:** 2015-04-24

**Authors:** Yusuke Tagashira, Tomoe Shimizu, Masanobu Miyamoto, Sho Nishida, Kaoru T. Yoshida

**Affiliations:** Graduate School of Agricultural and Life Sciences, The University of Tokyo, Bunkyo-ku, Tokyo 113-8657, Japan; E-Mails: tagashira0812@gmail.com (Y.T.); 1176hours@gmail.com (T.S.); masanyobu@yahoo.co.jp (M.M.); asho3@mail.ecc.u-tokyo.ac.jp (S.N.)

**Keywords:** ectopic expression, mineral element, molecular breeding, *Oryza sativa* L., phosphorus, phytic acid, seed, translocation

## Abstract

The manipulation of seed phosphorus is important for seedling growth and environmental P sustainability in agriculture. The mechanism of regulating P content in seed, however, is poorly understood. To study regulation of total P, we focused on phytic acid (inositol hexakisphosphate; InsP_6_) biosynthesis-related genes, as InsP_6_ is a major storage form of P in seeds. The rice (*Oryza sativa* L.) low phytic acid mutant *lpa1-1* has been identified as a homolog of archael 2-phosphoglycerate kinase. The homolog might act as an inositol monophosphate kinase, which catalyzes a key step in InsP_6_ biosynthesis. Overexpression of the homolog in transgenic rice resulted in a significant increase in total P content in seed, due to increases in InsP_6_ and inorganic phosphates. On the other hand, overexpression of genes that catalyze the first and last steps of InsP_6_ biosynthesis could not increase total P levels. From the experiments using developing seeds, it is suggested that the activation of InsP_6_ biosynthesis in both very early and very late periods of seed development increases the influx of P from vegetative organs into seeds. This is the first report from a study attempting to elevate the P levels of seed through a transgenic approach.

## 1. Introduction

Early development of seedlings is completely dependent on seed nutrient reserves. Seeds accumulate a large amount of phosphorus (P) to sustain seedling growth. Seeds store P mainly in the form of phytic acid (inositol hexakisphosphate; InsP_6_), with approximately 70% to 80% of total P stored in the form of InsP_6_ [1]. After imbibition, phytase hydrolyzes InsP_6_ in seeds, and the resulting available P is remobilized into shoots and roots.

Initial seedling growth is supported by available P in the seeds. As the plant develops, it proceeds from P-heterotrophy (P supply from seed) to P-autotrophy (uptake of external P via roots). In maize (*Zea mays* L.), the P-heterotrophic growth phase continues for 4 d after sowing, and the P-autotrophic phase starts after 16 d after sowing [2]. From 5 to 15 d after sowing, seedling growth is supported by both seed P and external P. The supply of nutrients from seed reserves to support early seedling development is, therefore, substantial. Ros *et al.* (1997) investigated the effect of seed P levels on early growth of rice and confirmed the beneficial effects from an increase in seed P content on plant growth and, in particular, the growth of roots [3].

In *Arabidopsis thaliana* L., reduced total P content was observed in seeds of the *atpap26* mutant, in which acid phosphatase activity was markedly reduced in the leaves. There was also a significant decrease in remobilization of P from old, senesced leaves to new leaves and to seeds [4]. Seed germination of the *atpap26* mutant was delayed. These results suggest that a reduction in total P content has a negative effect on seed performance. On the contrary, higher seed P content has led to more rapid seedling emergence and larger biomass in several species [5,6].

**Figure 1:**
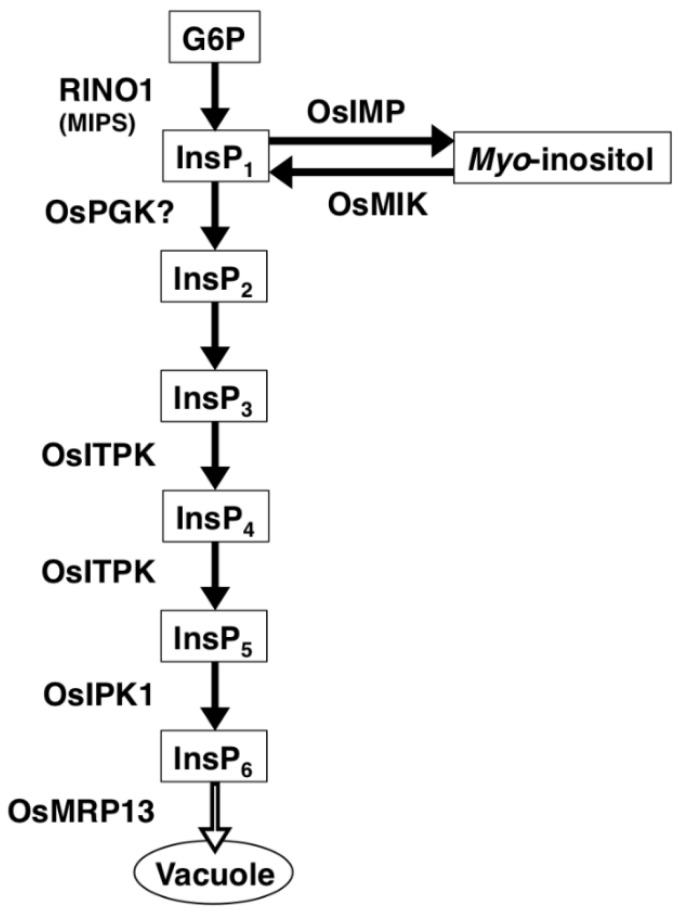
Scheme for biosynthesis of phytic acid (Ins P_6_) and inositol in rice.

Control of seed total P content is important not only for seed performance but also for environmental sustainability of P in agriculture [1]. However, the control mechanism of total P content in seed is poorly understood. The majority of seed P is stored in the form of InsP_6_, so it is plausible that total P content in seed might increase if the InsP_6_ content is increased. In fact, InsP_6_ and total P contents are closely correlated in beans [7,8]. To elevate the InsP_6_ level in a seed, it is important to activate the InsP_6_ biosynthetic pathway by increasing the expression level of the rate-limiting enzymes in that pathway. *Myo*-inositol 3-phosphate synthase (MIPS), which catalyzes the first step of InsP_6_ biosynthesis and inositol metabolism, has been considered as a key enzyme in these pathways [9] (Figure 1). There are two MIPS genes in the rice genome, *RINO1* (Os03g0192700) and *RINO2* (Os10g0369900). *RINO1* is responsible for InsP_6_ biosynthesis in rice seeds, because *RINO1* transcript levels are extremely high in developing seeds and *RINO2* mRNA is scarcely detected [10]. Another important step is the last step of InsP_6_ biosynthesis, from inositol pentaphosphate (InsP_5_) to InsP_6_, which is catalyzed by inositol 1,3,4,5,6-penta*kis*phosphate 2-kinase (IPK1) (Figure 1). The rice *IPK1* gene, *OsIPK1* (Os04g0661200), is highly expressed in developing seeds [10]. Activation of the first or last step of InsP_6_ biosynthesis might lead to activation of InsP_6_ biosynthesis and an increase in total P content in seeds.

The step from InsP_1_ to inositol diphosphate (InsP_2_) is another key step in the InsP_6_ biosynthetic pathway, as InsP_1_ lies at an important branch point between InsP_6_ biosynthesis and inositol metabolism (Figure 1). Kim *et al.* (2008) identified a rice low phytic acid mutant (*lpa1*) as a homolog of 2-phosphoglycerate kinase (2-PGK), which catalyzes the step from 2-phosphoglycerate to 2,3-bisphosphoglycerate in archaea [11]. From structural analogy of substrates and products, the protein encoded by rice *lpa1* might function as an InsP_1_ kinase, which catalyzes the step from InsP_1_ to InsP_2_ [1,11]. If the protein catalyzes the key branch point step of InsP_6_ biosynthesis, overexpression of the protein might lead to an increase in InsP_6_ accumulation in seeds.

In the rice genome, there are two putative 2-PGK homologs, which were identified by a BLAST search. We designated the homologs as OsPGK1 (Lpa1; Os02g0819400) and OsPGK2 (Os09g057220). Alignment of the deduced amino acid sequence of OsPGK1 indicated an approximately 60% similarity to OsPGK2. OsPGK1 might play a major role in phytic acid biosynthesis in developing seeds, because the mutation of this gene resulted in a severe lpa phenotype [11,12]. The expression pattern of *OsPGK1* is immature seed-specific, which is apparent from the microarray data in RiceXPro (http://ricexpro.dna.affrc.go.jp).

In this study, to increase InsP_6_ content in rice seeds, we generated transgenic rice plants that overexpressed the rice genes *RINO1*, *OsIPK1*, and *OsPGK1* under the control of an actin promoter. We designated the *RINO1*, *OsIPK1*, and *OsPGK1* overexpressors as RINO1ox, OsIPK1ox, and OsPGK1ox, respectively. We investigated InsP_6_ and total P content in seeds from these transgenic plants and discovered that only OsPGK1ox significantly increased InsP_6_ and total P contents in the seed, compared to the non-transformant (NT) seed. We discuss the causal mechanisms of these increases.

## 2. Results

### 2.1. The Effect of RINO1 or OsIPK1 Overexpression on InsP_6_ Accumulation in Seeds

**Figure 2:**
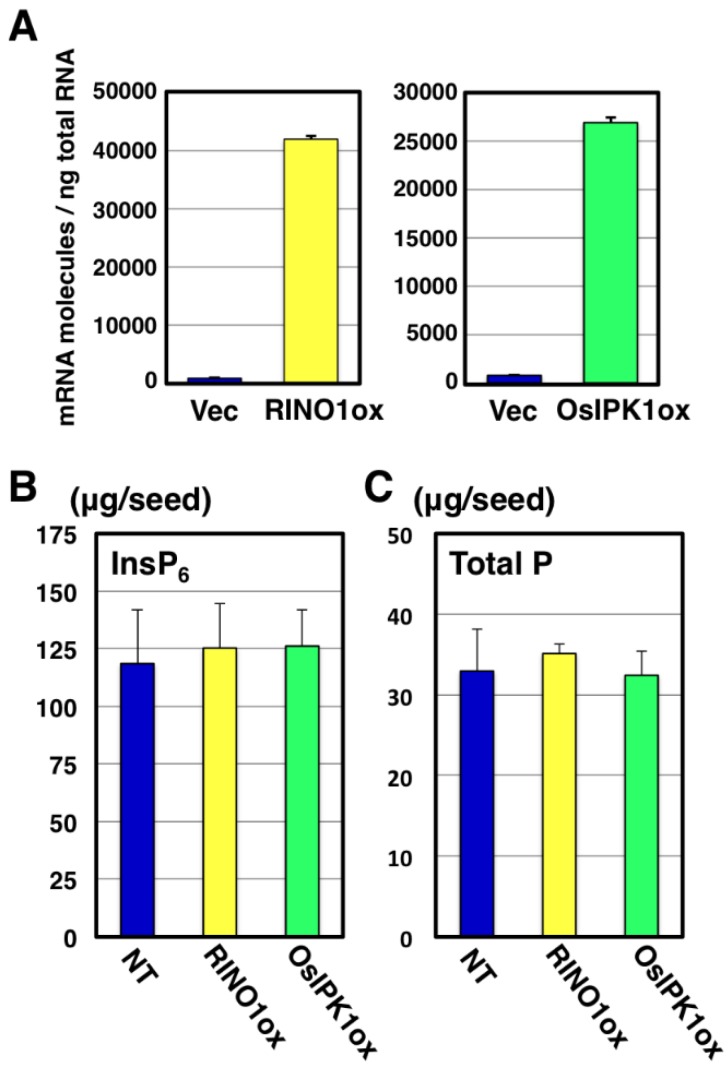
Gene expression and seed phenotype of RINO1ox and OsIPK1ox transgenic plants. (**A**) Quantitative RT-PCR analysis of *RINO1* (left) and *OsIPK1* (right) genes with cDNA templates from leaves of vector control (Vec) and RINO1ox or OsIPK1ox plants 7 d after germination (*n* = 3); (**B**) InsP_6_ content in mature seeds obtained from non-transformant (NT) and two transgenic plants was determined by ion chromatography (*n* ≥ 7); (**C**) Total P content in NT and two transgenic seeds was measured by colorimetric assay (*n* ≥ 5). Each value (**A** to **C**) represents the mean ± SD.

We first observed the seed phenotypes of RINO1ox and OsIPK1ox, as these enzymes have been well characterized in InsP_6_ biosynthesis [9,10]. From the three fixed progeny lines of each transformant, we selected a RINO1ox line and an OsIPK1ox line, based on their high expression in T_3_ plants (Figure 2A). InsP_6_ content in T_4_ seeds was measured by ion chromatography. InsP_6_ content in the RINO1ox and OsIPK1ox seeds was slightly higher than in NT seed, albeit not significantly so. (Figure 2B). Seed total P content in both transgenic plants was identical to that of NT seed (Figure 2C).

### 2.2. Seed Phenotype of the OsPGK1 Overexpressing Transformants

**Figure 3:**
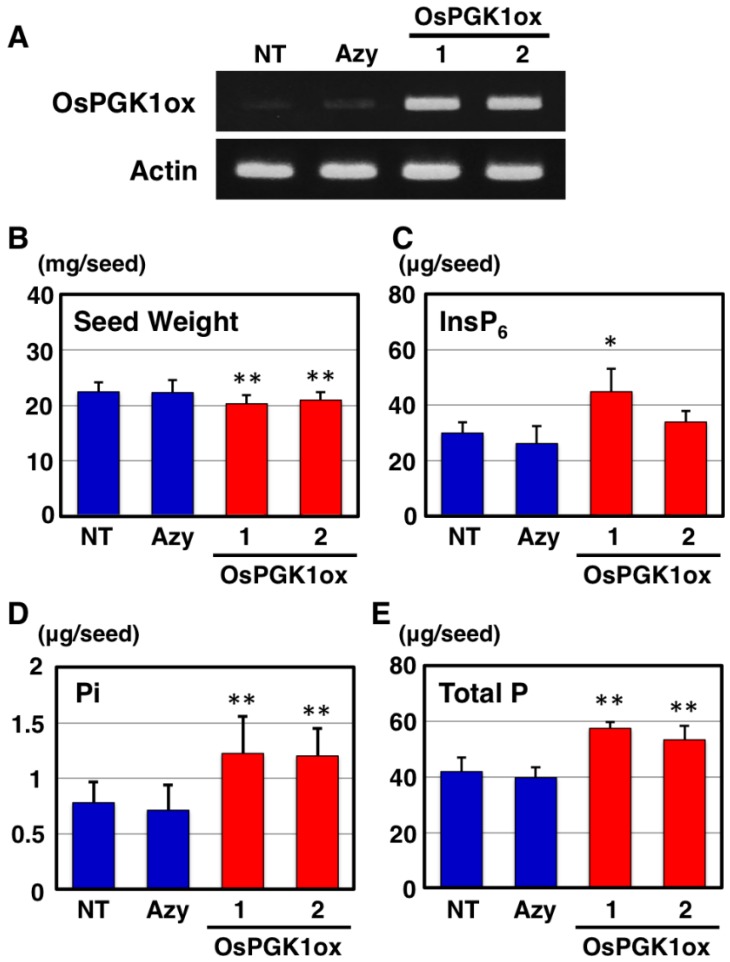
Gene expression and seed phenotype of OsPGK1ox transgenic plants. (**A**) Semi-quantitative RT-PCR analysis of the expression of *OsPGK1*. Total RNA was extracted from flowers of non-transformants (NT), azygous plants (Azy), and two independent OsPGK1ox lines (1 and 2) just before flowering. Actin was used as a reference; (**B**) Seed weights (*n* = 20); (**C**) InsP_6_ content was determined by ion chromatography (*n* = 4); (**D**) Inorganic phosphate content was measured by colorimetric assay using molybdate staining (*n* = 10); (**E**) Total P content was measured by ICP-OES analysis (*n* = 6). Each value (**B** to **E**) represents the mean ± SD. * and ** indicate *p* < 0.05 and *p* < 0.01, respectively.

As a next step, we generated transgenic rice plants carrying a rice *2-PGK* homolog gene (*OsPGK1*), driven by a rice actin promoter. Five primary transformants (OsPGK1ox) were obtained after regeneration from antibiotic-resistant calli. All plants produced viable T_1_ seeds. After measuring InsP_6_ content in T_1_ seeds, we selected two transgenic lines (OsPGK1ox-1 and OsPGK1ox-2), which exhibited increased seed InsP_6_ contents, and the fixed progeny lines were used in subsequent experiments. We confirmed overexpression of the *OsPGK1* gene in these transgenic lines (Figure 3A). We selected an azygous line (AZ), which was the transformed line without the transgene, derived from an OsPGK1ox-2 line.

We examined the phenotype of T_4_ seeds. Although the dried mature seed weights of the OsPGK1ox lines were significantly less than weights of NT and AZ seeds (Figure 3B), there were no significant differences between the NT and OsPGK1ox lines in terms of germination rate, early seedling growth, and plant height. There was a significant increase in total InsP_6_ content in the OsPGK1ox-1 seeds and a slight increase in total InsP_6_ content in the OsPGK1ox-2 seeds compared with NT and AZ seeds (Figure 3C). Contrary to expectations, Pi content also increased in the seeds of OsPGK1ox lines (Figure 3D). Seed total P content was significantly higher in both transgenic lines than in NT and AZ (Figure 3E). Total P content increased by 1.29-fold for OsPGK1ox-2 seeds and 1.37-fold for OsPGK1ox-1 seeds, compared with NT seeds.

### 2.3. Accumulation of InsP_6_ and Total Phosphorus in Developing Seeds of OsPGK1ox

**Figure 4:**
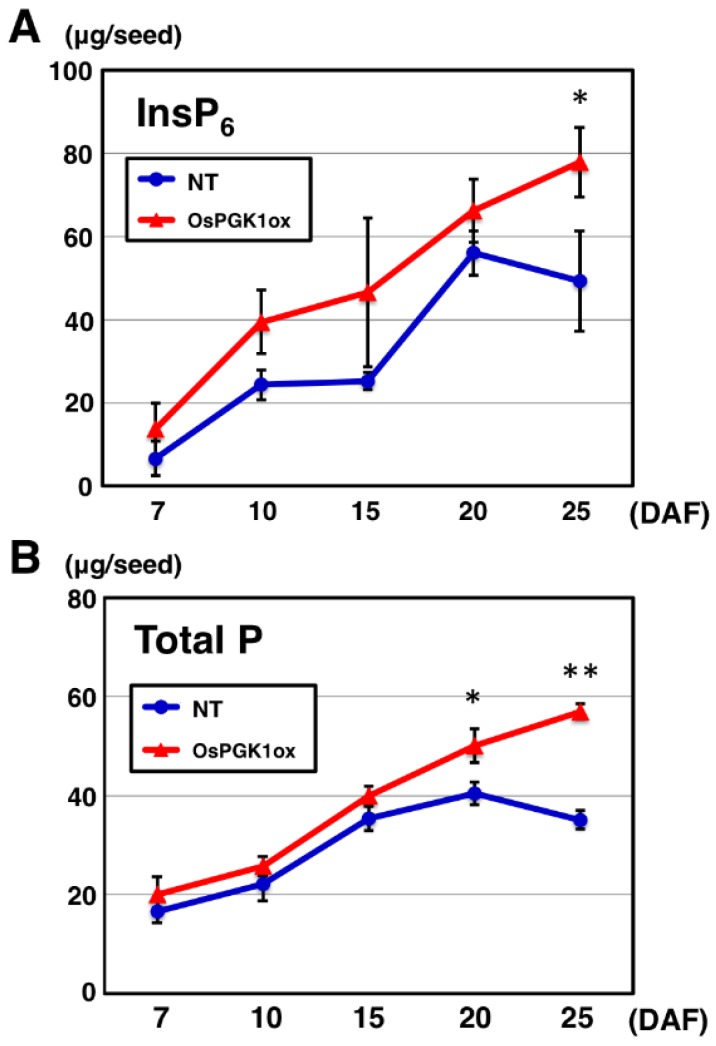
Changes in InsP_6_ (**A**) and total P (**B**) content in immature seeds of OsPGK1ox-1 and NT during seed development from 7 to 25 d after flowering (DAF). InsP_6_ and total P contents were determined by ion chromatography and ICP-OES analyses, respectively. Each value represents the mean ± SD of three replicates. * and ** indicate *p* < 0.05 and *p* < 0.01, respectively.

To determine changes in InsP_6_ content during seed development, seeds of OsPGK1ox-1 and NT were analyzed by ion chromatography at 7, 10, 15, 20, and 25 d after flowering (DAF; Figure 4A). InsP_6_ content in NT seeds increased from 7 to 20 DAF and stopped at 20 DAF. In contrast, the increase in InsP_6_ content in the OsPGK1ox-1 seeds continued from 7 to 25 DAF. InsP_6_ content in the OsPGK1ox-1 seeds at 25 DAF was significantly higher than InsP_6_ content in the NT seeds. Although InsP_6_ content in the OsPGK1ox-1 seeds was slightly higher than InsP_6_ content in the NT seeds from 7 to 20 DAF, the rates of InsP_6_ content increase were similar for the OsPGK1ox-1 and NT seeds during this period (Figure 4A).

Similar results were obtained for total P content in developing seeds analyzed by inductively coupled plasma optical-emission spectrometry (ICP-OES; Figure 4B). Total P content in NT seeds rapidly increased until 15 DAF, and then gradually increased between 15 and 20 DAF. The increase in total P content stopped at 20 DAF in the NT seeds. In contrast, the increase in total P content in the OsPGK1ox-1 seeds continued from 7 to 25 DAF. Total P content in the OsPGK1ox-1 seeds at 20 and 25 DAF was significantly higher than total P content in the NT seeds. These results indicate that an influx of P from vegetative organs to seeds continued after 20 DAF only in the OsPGK1ox-1 plants. Although total P content in the OsPGK1ox-1 line, from 7 to 20 DAF, was slightly higher than total P content in the NT line, the rates of increase in total P were similar for OsPGK1ox-1 and NT seeds during this period (Figure 4B).

### 2.4. Mineral Contents in the Seeds of OsPGK1ox

**Figure 5:**
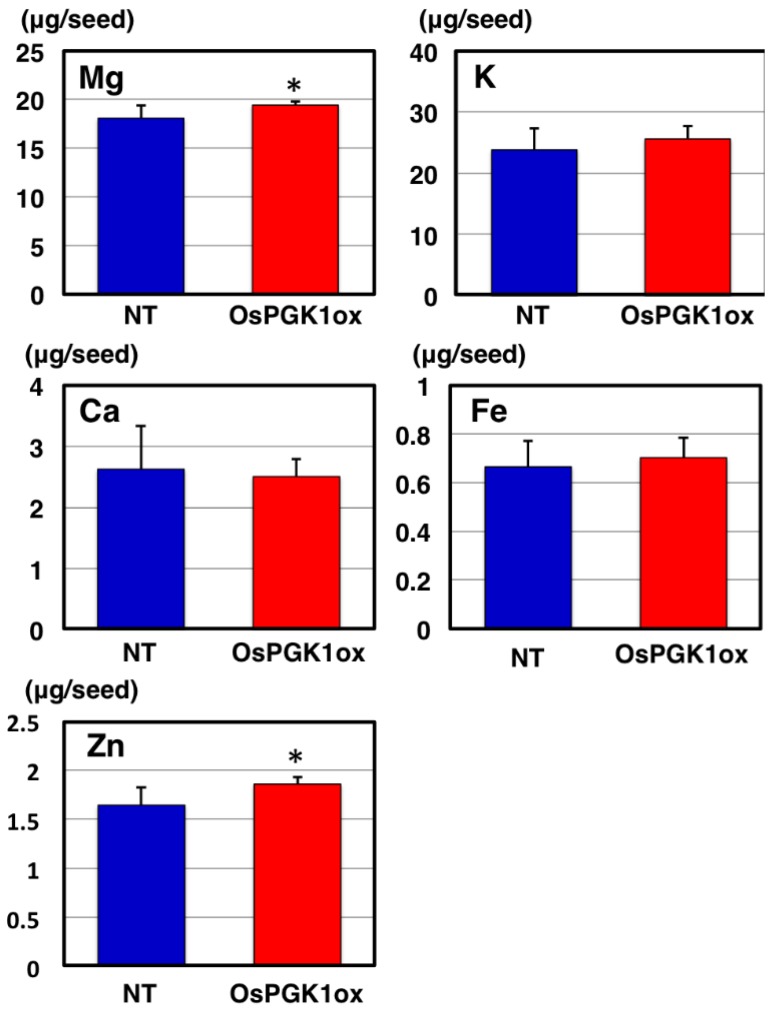
Contents of Mg, K, Ca, Fe, and Zn in mature seeds of OsPGK1ox and NT. The mineral contents were measured by ICP-OES analysis. The analysis in Figure 3E and Figure 5 was performed simultaneously using the same seed samples. Each value represents the mean ± SD of six replicates. * indicates *p* < 0.05.

In OsPGK1ox plants, a reduced number of grains per panicle was observed compared to NT panicles. The reductions were 40.4% for OsPGK1ox-1 and 27.1% for OsPGK1ox-2. We measured the contents of several mineral elements (Mg, K, Ca, Fe, and Zn) in OsPGK1ox-1 seeds, using ICP-OES analysis, to determine if the decrease in grains per panicle affected mineral influx from vegetative organs into seeds (Figure 5). Calcium was the negative control, because the influx of Ca into a seed via the phloem is limited during seed development [13]. The OsPGK1ox seeds tended to exhibit increased contents of mineral elements, except for Ca, as compared with NT seeds (Figure 5). Contents of mineral elements in the OsPGK1ox seeds increased by 1.07-fold for Mg and K, 1.05-fold for Fe, and 1.13-fold for Zn, compared to the NT seeds. However, total P content in the same OsPGK1ox-1 seeds was 1.37-fold higher than total P content in the NT seeds (Figure 3E).

### 2.5. Expression of Phytic Acid Biosynthesis-related Genes in OsPGK1ox

**Figure 6:**
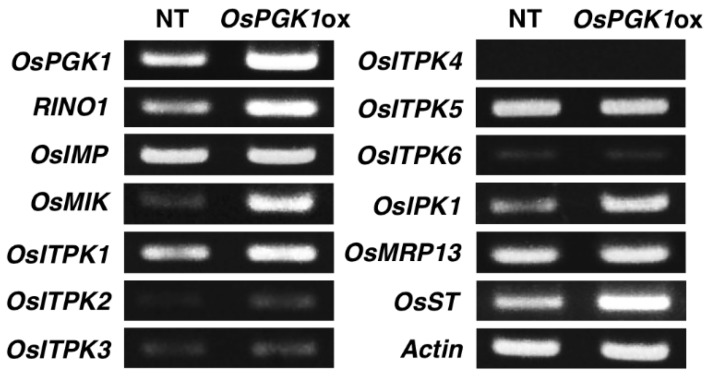
Semi-quantitative RT-PCR analysis of the expression of 13 phytic acid biosynthesis-related genes in the OsPGK1ox and NT plants. Total RNA was extracted from the roots of non-transformant (NT) and OsPGK1ox-1 3 d after germination. Actin was used as a reference.

To determine whether the overexpression of *OsPGK1* affects expression levels of other phytic acid-biosynthesis related genes, we analyzed the expression of several genes (*RINO1*, *OsIMP*, *OsMIK*, *OsITPK*, *OsIPK1*, *OsMRP13*, and *OsST*; Figure 1) by RT-PCR (Figure 6). OsIMP is a *myo*-inositol monophosphatase, OsMIK is a *myo*-inositol kinase, and OsITPK is an inositol 1,3,4-tris*kis*phosphate 5/6-kinase. OsMRP13/OsABCC13 is an ortholog of AtMRP5, which is a putative transporter of InsP_6_ [14]. OsST is an ortholog of barley (*Hordeum vulgare* L.) HvST, which is a member of the sulfate transporter gene family and is the causal gene of a barley *lpa* mutant [15].

In our previous studies, real-time RT-PCR analysis of mRNA levels revealed strong up-regulation of the phytic acid-biosynthesis related genes (e.g., *RINO1*, *OsIPK1*, and *OsITPK*) in immature rice seeds. The expression levels of these genes were more than tenfold greater in seeds than in vegetative tissues [10]. It is difficult to detect changes in the expression levels of the genes using analysis of total RNA extracted from immature seeds. To detect transcript level changes induced by overexpression of *OsPGK1*, we used total RNA extracted from roots as RT-PCR templates. InsP_6_ in roots could be detected by ion chromatography even if the InsP_6_ content was 1% of that of seeds (data not shown). Therefore, we concluded that the InsP_6_ synthetic pathway is also active in roots, even though activity was lower in roots than in seeds.

To determine the effect of overexpression of *OsPGK1* on expression of the other genes, we used total RNA extracted from roots for RT-PCR analysis. Among phytic acid biosynthesis-related genes, expression of the *OsMIK*, *OsITPK2*, *OsITPK3*, *OsITPK4*, and *OsITPK6* genes was barely detected in the NT roots (Figure 6). However, expression of *OsMIK* gene was markedly induced, and expression of *OsITPK2* and *OsITPK3* genes was slightly induced in OsPGK1ox roots. Additionally, the expression levels of *RINO1*, *OsITPK1*, and *OsIPK1* in OsPGK1ox were higher than in the NT (Figure 6). The expression of *OsST* was also higher in OsPGK1ox than in NT, although we do not know its function in InsP_6_ biosynthesis. The expression levels of transcripts of *OsIMP* and a putative phytic acid transporter, *OsMRP13*, displayed similar differences between OsPGK1ox and NT.

## 3. Discussion

There have been many reports on *lpa* mutants and on lpa transgenic plants [1,16]. In *lpa* seeds, the reduction in InsP_6_ content is often accompanied by a molar-equivalent increase in Pi content, so total P content is almost unchanged. There are, however, two *lpa* mutants that have a reduction in total P content. Arabidopsis *atmrp5*, a putative transporter for InsP_6_, reduced total P content by 35% [14], and barley *lpa1-1/hvst*, a homolog of a sulfate transporter, reduced total P content by 15% [15,17]. There have been no reports on mutants or transgenic plants with increases in InsP_6_ or total P content. To our knowledge, this is the first report of significant increases in both InsP_6_ and total P contents in seeds.

Overexpression of *RINO1* or *OsIPK1* was not effective in activating InsP_6_ biosynthesis (Figure 2). In the RINO1ox plants, accumulation of InsP_1_ and its dephosphorylated product, inositol, has been observed [18]. This suggests that InsP_1_ is rapidly dephosphorylated into inositol in RINO1ox plants (Figure 1), which might explain the limited increase in InsP_6_ in RINO1ox seeds (Figure 2). For OsIPK1ox, it is possible that there is a rate-limiting step before the last step of InsP_6_ biosynthesis, which is catalyzed by OsIPK1, and, therefore, overexpression of *OsIPK1* has a very slight effect on activation of InsP_6_ biosynthesis. We do not know if expression of InsP_6_ biosynthesis-related genes is induced in both RINO1ox and OsIPK1ox lines. In any case, the fact that only the overexpression of *OsPGK1* led to activation of InsP_6_ biosynthesis suggests that *OsPGK1* is a key gene for InsP_6_ synthesis and that OsPGK1 is involved in the branch and, probably, the rate-limiting step from InsP_1_ to InsP_2_.

From the RT-PCR analysis results, we revealed that the expression of many phytic acid biosynthesis-related genes increased in OsPGK1ox plants (Figure 6). Among these genes, the expression of *OsMIK*, *myo*-inositol kinase, which catalyzes the step from *myo*-inositol to InsP_1_, was markedly induced in OsPGK1ox plants. If OsPGK1 is involved in the branch step from InsP_1_ to InsP_2_, overexpression of *OsPGK1* might lead to a deficiency in InsP_1_. It is plausible that the expression of *OsMIK* and *RINO1* might be raised in OsPGK1ox to compensate for the InsP_1_ deficiency.

The rates of InsP_6_ content increase, from 7 to 20 DAF, were similar for the OsPGK1ox and NT seeds (Figure 4A). This indicated that the effect of overexpression of *OsPGK1* was barely observed from 7 to 20 DAF. In contrast, after 20 DAF, synthesis of InsP_6_ continued in the OsPGK1ox seed, but not in the NT seed (Figure 4A). Also, InsP_6_ content in the OsPGK1ox seed was higher than that in NT seed at 7 DAF, although the difference was not significant.

Expression levels of the InsP_6_ biosynthesis-related genes are usually highest between 7 and 21 DAF [10]. Microarray analysis revealed that the level of transcripts in *OsPGK1* seed is also high during that period, but not during the other periods (RiceXPro). It is plausible that the overexpression of *OsPGK1* was very effective in activating InsP_6_ biosynthesis before 7 DAF and after 20 DAF, which explained the increase in InsP_6_ content in OsPGK1ox seeds at very early and very late periods (Figure 4A). We plan to compare the gene expression levels in OsPGK1ox and NT seeds before 7 DAF and after 20 DAF in future studies.

In this study, the increase in InsP_6_ content was accompanied by an increase in total P content. In the OsPGK1ox line, the increase in total P content continued after 20 DAF and total P content at 7 DAF was higher than total P content in the NT line, as was the case for InsP_6_. This indicates that influx of P from vegetative organs into seeds is more active in OsPGK1ox, and the activity of InsP_6_ biosynthesis affects the influx of P. The signal for determining the amount of P influx and involvement of OsPGK1 in P influx has not been elucidated. In Arabidopsis, AtPAP26 encoding purple acid phosphatase is up-regulated during leaf senescence and the *atpap26* mutant displayed delayed leaf senescence and reduced seed total P content [4]. This indicates that AtPAP26 plays a key role in remobilization of P from old leaves to seeds. Waters and Grusak (2008) investigated quantitative trait loci (QTL) that control seed P concentration in two Arabidopsis recombinant inbred line populations [19]. Some phosphate transporters belonging to the phosphate transporter 1 (Pht1) family were listed as candidate genes in the QTL regions [19]. The Pht1 Pi transporters are active in the uptake of Pi from the soil and its translocation within the plant, and some transporters might affect P remobilization from vegetative organs into seeds. Attention should be paid to both the phosphatases and the Pi transporters that are specific for P remobilization.

In this study, the number of grains per panicle was greatly reduced in the OsPGK1ox plant. The level of *myo*-inositol in the OsPGK1ox cells might be lower than NT cells, because the overexpression of *OsPGK1* induced the high expression of *OsMIK* gene (Figure 1 and Figure 6). *myo*-Inositol is a central compound in diverse biochemical processes, including signal transduction, stress protection, cell wall biogenesis, growth regulation, and membrane trafficking [20]. Therefore, it is possible that a decrease of *myo*-inositol level in shoot apical meristems caused by ectopic expression of *OsPGK1* leads to an alteration in the development of inflorescences and floral organs in the OsPGK1ox plant. This might result in the reduced number of grains per panicle.

The reduced number of grains per panicle might influence the translocation of mineral elements. However, contents of mineral elements other than P increased only slightly in the OsPGK1ox line (Figure 5). Therefore, the large increase in P content in the seeds of OsPGK1ox was mainly due to the specific response to overexpression of *OsPGK1*, not to the reduced number of grains per panicle. We plan to attempt to generate transgenic plants that overexpress *OsPGK1* under the control of a seed specific promoter, such as the 18-kDa oleosin promoter, which promotes expression in the embryo and aleurone layer, where phytic acid is synthesized [16].

Many reports have suggested that seed P content has a beneficial effect on seed performance, in terms of germination rate and early seedling growth [3,4,5,6]. However, OsPGK1ox seed performance was similar to that of NT seed, although seed total P content in OsPGK1ox increased by 1.3 to 1.4-fold. Further study is needed to determine the reason for identical seed performance of the OsPKG1ox and NT plants.

Control of the total P content of seeds is important to enhance P sustainability and decrease the environmental impacts of agriculture [1]. We hope that this report provides the first step toward manipulating seed total P.

## 4. Experimental Section

### 4.1. Transformation of Rice Plants

*OsIPK1* or *OsPGK1* cDNA were amplified by RT-PCR using total RNA prepared from immature seeds (cv. Nipponbare) at 10 DAF. The gene-specific primer pairs were 5'-CTGATTCTGTGTGGGGATGG-3' and 5'-AAATTCGGCCTACTGCTGAG-3' for *OsIPK1*, and 5'-GGGAGGCCTCTTCTTGATTC-3' and 5'-TTGACACCGGAGGCACTATG-3' for *OsPGK1*. Amplified cDNA with a length of 1578 bp for *OsIPK1* or 2523 bp for *OsPGK1* was cloned into the binary vector pBIAct/nos containing the rice *Actin1* promoter [21] and *nos* terminator. The method of plasmid construction was similar to that described previously [18]. Transgenic rice (*Oryza sativa* L., cv. Kitaake) was produced by the *Agrobacterium*-mediated method and grown in a glasshouse. The method to produce *RINO1*-overexpressing rice was described previously [18].

The presence of introduced genes was confirmed by PCR using specific primer sets for the *Act1* promoter (5'-TCCCTCAGCATTGTTCATCG-3') and the *RINO1* (5'-ACCAGCTCCGTCGTGTCGTA-3'), *OsIPK1* (5'-GGTGCCGGTTGTCCCTTGTC-3'), and *OsPGK1* (5'-GCCTTGCATCCCATGAGTTG-3') genes.

### 4.2. Measurements of Seed Components

InsP_6_ content in T_4_ seeds was measured by ion-chromatography and the Pi levels of T_4_ seeds were measured by molybdate-staining assay. The detailed method of Pi and InsP_6_ measurements was described previously [22]. Total P content was determined by either colorimetric assay or by inductively coupled plasma optical-emission spectrometry (ICP-OES) analysis. The detailed methods using the colorimetric assay were described in Kuwano *et al.* (2009) [16]. The methods of ICP-OES analysis of total P and other minerals (Mg, K, Ca, Fe, and Zn) were described previously [23]. Before analysis, the developing T_4_ seeds at 7, 10, 15, 20, 25 DAF were vacuum-freeze dried for 2 d and mature seeds were dried for 2 d at 60 °C.

### 4.3. Expression Analysis

Quantitative RT-PCR analysis of *RINO1* and *OsIPK1* genes was performed with cDNA templates from leaves of vector control plants carrying the empty vector, RINO1ox (T_3_), or OsIPK1ox (T_3_) plants at 7 d after germination. The detailed methods were described in Suzuki *et al.* (2007) [10].

For RT-PCR analysis, total RNA was prepared from flowers just before flowering or roots at 3 d after germination of NT (cv. Kitaake) and OsPGK1ox plants. The detailed methods of RT-PCR analysis were described previously [23]. The *Actin* gene was used as the control. The gene-specific PCR primer sets are listed in Table S1. The experiment was repeated three times with three biologically independent RNA samples.

## 5. Conclusions

To regulate total P in seeds, we focused on InsP_6_ biosynthesis-related genes because InsP_6_ is a major storage form of P in seeds. We generated transgenic rice plants that overexpressed InsP_6_-biosynthesis key genes, *RINO1*, *OsIPK1*, and *OsPGK1*. Only the overexpression of *OsPGK1* resulted in a significant increase in seed total P, due to the increases in InsP_6_ and Pi. It is strongly suggested that the overexpression of *OsPGK1* may lead the increase in influx of P from vegetative organs into seeds and may activate InsP_6_ biosynthesis. This is a first report to elevate the seed P levels through a transgenic approach.

## Supplementary Files

Supplementary File 1
