# Supplementary material for: Overexpression of a Gene Involved in Phytic Acid Biosynthesis Substantially Increases Phytic Acid and Total Phosphorus in Rice Seeds
Source: Plants (Basel). 2015 Apr 24;4(2):196–208. doi: 10.3390/plants4020196 (PMC4844318; doi:10.3390/plants4020196)
Supplement: Supplementary File 1 [file plants-04-00196-s001.pdf]

Supplementary Material

Table S1. List of primers for RT-PCR analysis.

Gene	RAP ID	Primer Sequences		Amplicon
		Forword	Reverse	Size(bp)
OsPGK1	Os02g0819400	CGCTTCTTGCCTTGCTTTGA	GCCTTGCATCCCATGAGTTG	212
RINO1	Os03g0192700	CCGTGGCATGTGGCAAAGAG	TGCATAGCCCGATAAGAGTC	132
OsMIK	Os03g0737700	TCCATCCATGGCGCCCTCTC	TCCACCTCAACCACCACTTC	75
OsIMP	Os03g0587000	ATCTGATGGCACGAAGAATG	CAATGCCATACTGCGTCCAA	218
OsITPK1	Os10g0103800	TCCTACTCAGCCTTGTGC	TCAATGGTATCACTCTCG	92
OsITPK2	Os03g0230500	TTCACCGATTTCTTGCTG	TGACTGCTAGACCGTTAC	119
OsITPK3	Os03g0726200	TACAAACGACGACCAAGC	TTACCACGGAGGATTTGC	136
OsITPK4	Os02g0466400	ACAAGACCTGTTCCTGAGCA	CGAGCTGACGGGCCAACACC	157
OsITPK5	Os10g0576100	CGAGGTACCAGGTATGCT	TCTCTTGGCTGCTCTAAC	238
OsITPK6	Os09g0518700	GAAGCAGATGACACAAAC	CTAAGTGACAAGCAACCT	142
OsIPK1	Os04g0661200	GTGTCTGTTGTATCTTGGTG	AAATTCGGCCTACTGCTGAG	155
OsMRP13	Os03g0142800	GGAGCACTACCAAGATAAGC	ATTGGTGAACTCCAGAAAAC	213
OsST	Os04g0652400	ATACAACGAGCGAATGATGC	GCTGAGAGAACAGGGTGGAA	198
Actin	Os03g0836000	GAACAACTGGGACGACAT	CCTTTGGGTTCAGAGGAG	113

© 2015 by the authors; licensee MDPI, Basel, Switzerland. This article is an open access article distributed under the terms and conditions of the Creative Commons Attribution license (http://creativecommons.org/licenses/by/4.0/).
